# Identification and Functional Annotation of Genome-Wide ER-Regulated Genes in Breast Cancer Based on ChIP-Seq Data

**DOI:** 10.1155/2012/568950

**Published:** 2012-12-31

**Authors:** Min Ding, Haiyun Wang, Jiajia Chen, Bairong Shen, Zhonghua Xu

**Affiliations:** ^1^Department of Viral and Gene Therapy, Eastern Hepatobiliary Surgery Hospital, Second Military Medical University, Shanghai 200438, China; ^2^School of Life science and Technology, Tongji University, Shanghai 200092, China; ^3^Center for Systems Biology, Soochow University, Suzhou Jiangsu 215006, China; ^4^Department of Cardiothoracic Surgery, Second Affiliated Hospital of Soochow University, Suzhou Jiangsu 215004, China

## Abstract

Estrogen receptor (ER) is a crucial molecule symbol of breast cancer. Molecular interactions between ER complexes and DNA regulate the expression of genes responsible for cancer cell phenotypes. However, the positions and mechanisms of the ER binding with downstream gene targets are far from being fully understood. ChIP-Seq is an important assay for the genome-wide study of protein-DNA interactions. In this paper, we explored the genome-wide chromatin localization of ER-DNA binding regions by analyzing ChIP-Seq data from MCF-7 breast cancer cell line. By integrating three peak detection algorithms and two datasets, we localized 933 ER binding sites, 92% among which were located far away from promoters, suggesting long-range control by ER. Moreover, 489 genes in the vicinity of ER binding sites were identified as estrogen response elements by comparison with expression data. In addition, 836 single nucleotide polymorphisms (SNPs) in or near 157 ER-regulated genes were found in the vicinity of ER binding sites. Furthermore, we annotated the function of the nearest-neighbor genes of these binding sites using Gene Ontology (GO), KEGG, and GeneGo pathway databases. The results revealed novel ER-regulated genes pathways for further experimental validation. ER was found to affect every developed stage of breast cancer by regulating genes related to the development, progression, and metastasis. This study provides a deeper understanding of the regulatory mechanisms of ER and its associated genes.

## 1. Introduction

Breast cancer is a complex disease with high occurrence. It involves a wide range of pathological entities with diverse clinical courses. Gene and protein expression have been extensively profiled in different subtypes of breast cancer [1]. Growth of human breast cells is closely regulated by hormone receptors. Estrogen receptor (ER), a hormonal transcription factor, plays a critical role in the development of breast cancer. Combined with estrogen, it regulates the expression of multiple genes. Studies have found that ER-positive and ER-negative breast cancers are fundamentally different [2]. The outcome of hormone receptor positive tumors is better than hormone receptor negative tumors [3]. Thus, the identification of ER target genes may reveal critical biomarkers for cancer aggressiveness and is therefore crucial to understanding the global molecular mechanisms of ER in breast cancer. To identify direct target genes of ER, it is necessary to map the ER binding sites across the genome. ChIP-Seq is an effective technology for the genome-wide localization of histone modification and transcription factor binding sites. It enables researchers to fully understand many biological processes and disease states, including transcriptional regulation of ES cells, tissue samples, and cancer cells. 

**Figure 1:**
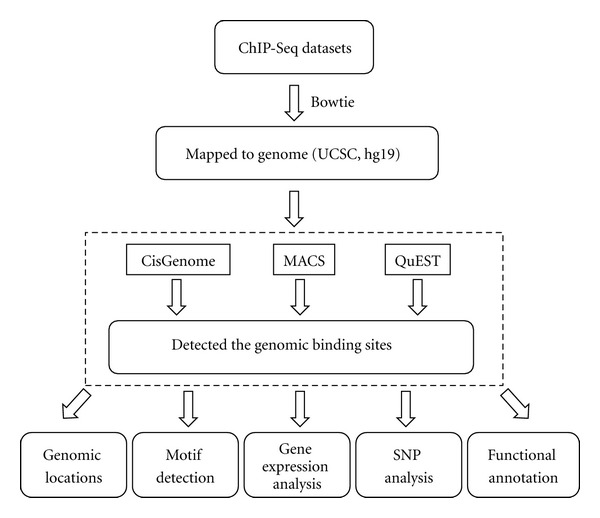
The ChIP-Seq data analyzing pipeline.

Several previous studies have been dedicated to ER-regulated genes and their function in breast cancer cell line [4, 5]. However, most studies lacked the comprehensive and genome-wide view and failed to perform an integrated analysis. In this study, we combined ChIP-Seq and microarray datasets to analyze the ER-regulated genes in the MCF-7 breast cancer cell line. The molecular mechanisms of ER were fully studied, including binding sites, motif, regulated genes, related single nucleotide polymorphisms (SNPs) and functional annotation. The process of this analysis was illustrated in Figure 1.

## 2. Materials and Methods

### 2.1. Datasets

The breast cancer associated ChIP-Seq datasets were extracted from Gene Expression Omnibus (GEO): GSE19013 [6] and GSE14664 [7]. Both datasets can be used to survey genome-wide binding of estrogen receptor (ER) in the MCF-7 breast cancer cell line. Control sample was incorporated for the genomic peak finding of ER. (See Table 1 for details.)

### 2.2. Chip-Seq Analysis

Bowtie [8] was selected to align sequence tags to human genome. Bowtie is an ultrafast and best short-read aligner. It is suitable for sets of short reads where many reads have at least one good and valid alignment, many reads with relatively high quality, and the number of alignment reported per read is small (closed to 1). ChIP-seq datasets we used were satisfied these criteria. In the analysis, tags were selected using the criterion that alignments had no more than 2 mismatches in the first 35 bases on the high quality end of the read, and the sum of the quality values at all mismatched positions could not exceed 70.

Peak detection algorithm is crucial to the analysis of ChIP-Seq dataset. Currently, several tools are available to identify genome-wide binding sites of transcription factors, such as FindPeaks [9], F-Seq [10], CisGenome [11], MACS [12], SISSRs [13], and QuEST [14]. These different methods have their own advantages and disadvantages, although they act in a similar manner.  Table 2 showed an overview of the characteristics of these algorithms. ChIP-Seq data has regional biases because of sequencing and mapping biases, chromatin structure, and genome copy number variations [15]. It is believed that more robust ChIP-Seq peak predictions can be obtained by matching control samples [12]. In order to get more stable result, three tools, CisGenome, MACS, and QuEST, were used to identify the binding sites of ER in this study. All the three tools systematically used control samples to guide peak finding and calculate the FDR (False Discovery Rate) value of peaks.

Additionally, MEME program [16] was employed for de novo motif search, keeping default options (minimum width: 6, maximum width: 50, motifs to find: 3, and minimum sites: ≥2). For each site, statistical significance (*P* value) gives the probability of a random string having the same match score or higher. And a criterion of *P*-value < 0.01 was used here.

### 2.3. Expression and SNP Analysis

Expression analysis was performed using the same package [17, 18]. Differentially expressed genes were selected based on the q-value less than 1%.

Using the table SNP (131) (dbSNP build 131) [19] in UCSC (http://genome.ucsc.edu/), we identified SNPs near the ER binding sites. The SNPs with at least one mapping in the regions were selected.

### 2.4. Functional Annotation

Three functional annotation systems, the Gene Ontology (GO) categories [20], canonical KEGG Pathway Maps [21], and commercial software MetaCore-GeneGo Pathway Maps, were used to perform the enrichment analysis for gene function. 

Enrichment of GO categories was determined with the Gene Ontology Tree Machine (GOTM) [22], using Hypergeometric test, Multiple test adjustment (BH), and a *P*-value cut-off of 0.01. WebGestalt (WEB-based GEne SeT AnaLysis Toolkit) [23] (http://bioinfo.vanderbilt.edu/webgestalt/option.php) was used for enrichment of KEGG Pathway. Hypergeometric test, Multiple test adjustment (BH), and a *P*-value cut-off of 0.01 were also used as criterion. MetaCore-GeneGo is a commercial software which offers gene expression pathway analysis and bioinformatics solutions for systems biology research and development. Hypergeometric intersection was used to estimate *P*-value, the lower *P*-value means higher relevance. *P*-value < 0.01 and FDR < 0.05 were used as criterion.

## 3. Results and Discussion

### 3.1. ChIP-Seq Analysis Mapped ER Binding Sites across the Human Genome

**Figure 2:**
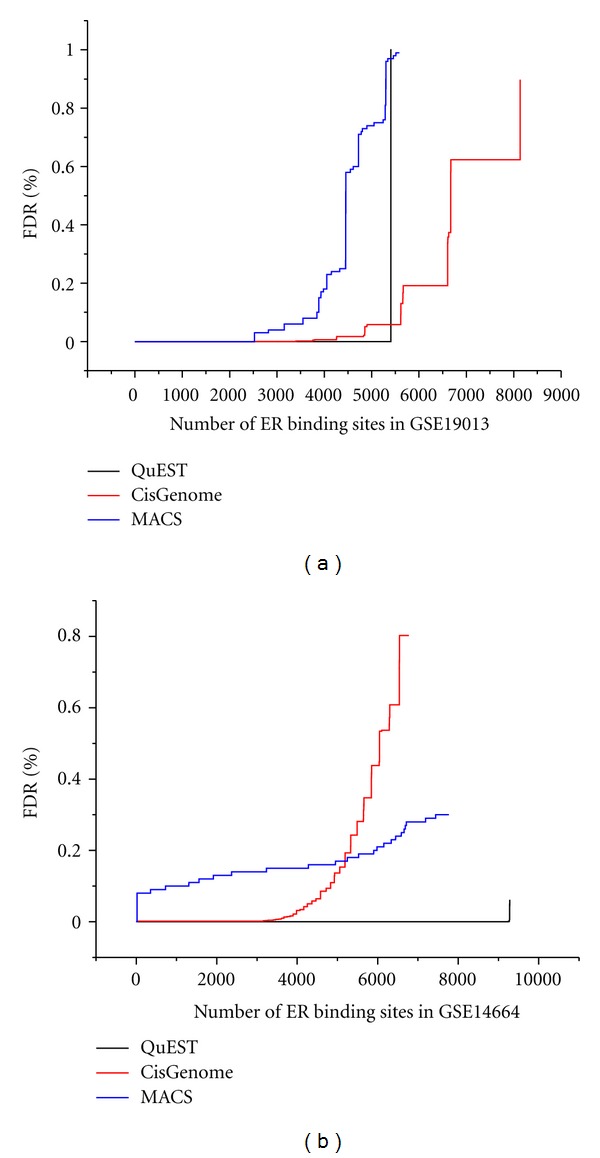
Comparison of QuEST, CisGenome, and MACS predicted result. (a) The FDR value in the dataset of GSE19013. (b) The FDR value in the dataset of GSE14664.

**Figure 3:**
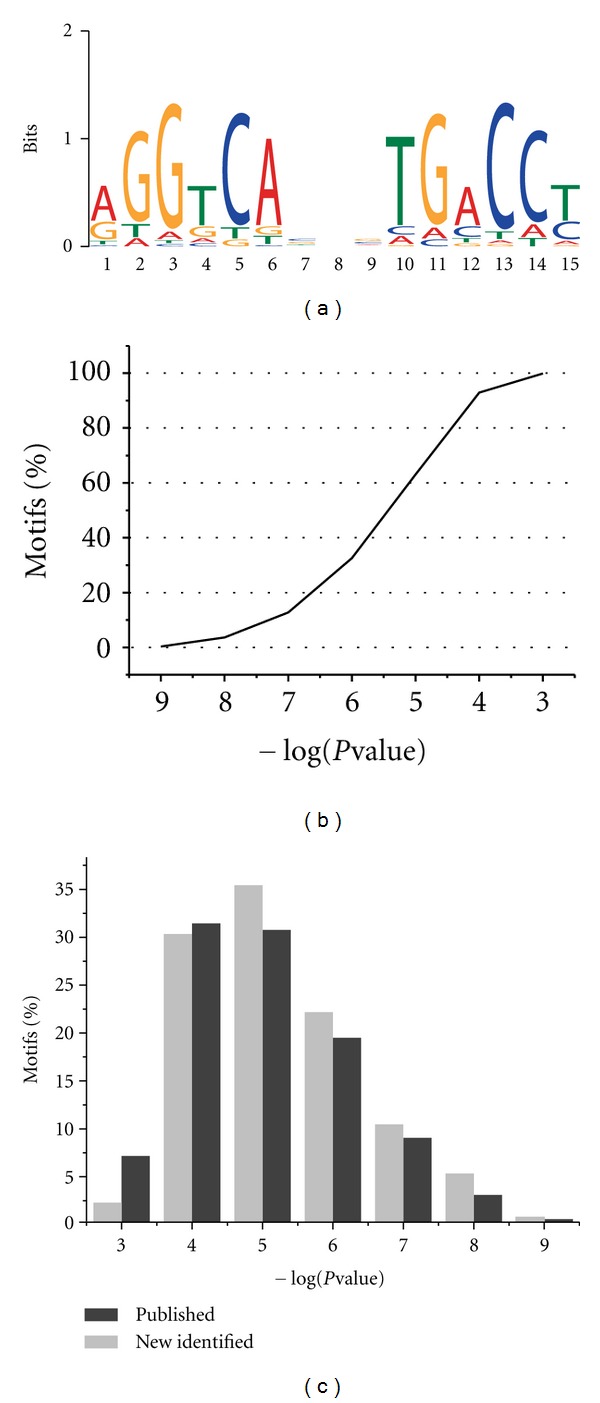
The genomic binding sites of ER. (a) The consensus motif identified in the ERE binding sites. De novo motif search was performed using the MEME program. (b) The percentage of occurrences of ERE motifs in ER binding sites. (c) Comparison of the occurrences of ERE motifs between published and newly identified binding sites.

 Using ChIP-Seq datasets, we identified the global ER binding sites. Sequence tags were firstly aligned to human genome assembly (UCSC, hg19) using Bowtie. Three ChIP-Seq peak calling programs, CisGenome, MACS, and QuEST, were selected to identify the enriched binding peaks. Using a false discovery rate of 0.01, 933 ER binding peaks were revealed by all the three tools in both datasets (Table 3). There were differences among the predicted results using different methods in both two datasets (Figure 2). The calculated FDR value was not only related to different methods, but also influenced by datasets. The overlapped binding sites seemed to be more robust, with 84.9% having FDR value less than 0.005 in all methods and datasets. These binding sites were used for the following analysis. Firstly, we compared these binding sites with two published studies by Welboren et al. [7] and Hu et al. [6]. Our results showed a substantial overlap with the two studies (77.8 and 78.5%, resp.). Also, 719 binding sites, which were shared by all three studies, were likely to be more reliable. The presence of consensus sequence motifs in the ER binding sites was also examined. De novo motif search using the MEME program [16] identified a refined ERE motif that was markedly similar to the canonical ERE (Figure 3(a)). Almost all of the ER binding sites contained one or more ERE motif (*P*-value < 0.01) (Figure 3(b)). Both published and newly identified binding sites contained at least one ERE motif (Figure 3(c)).

**Figure 4:**
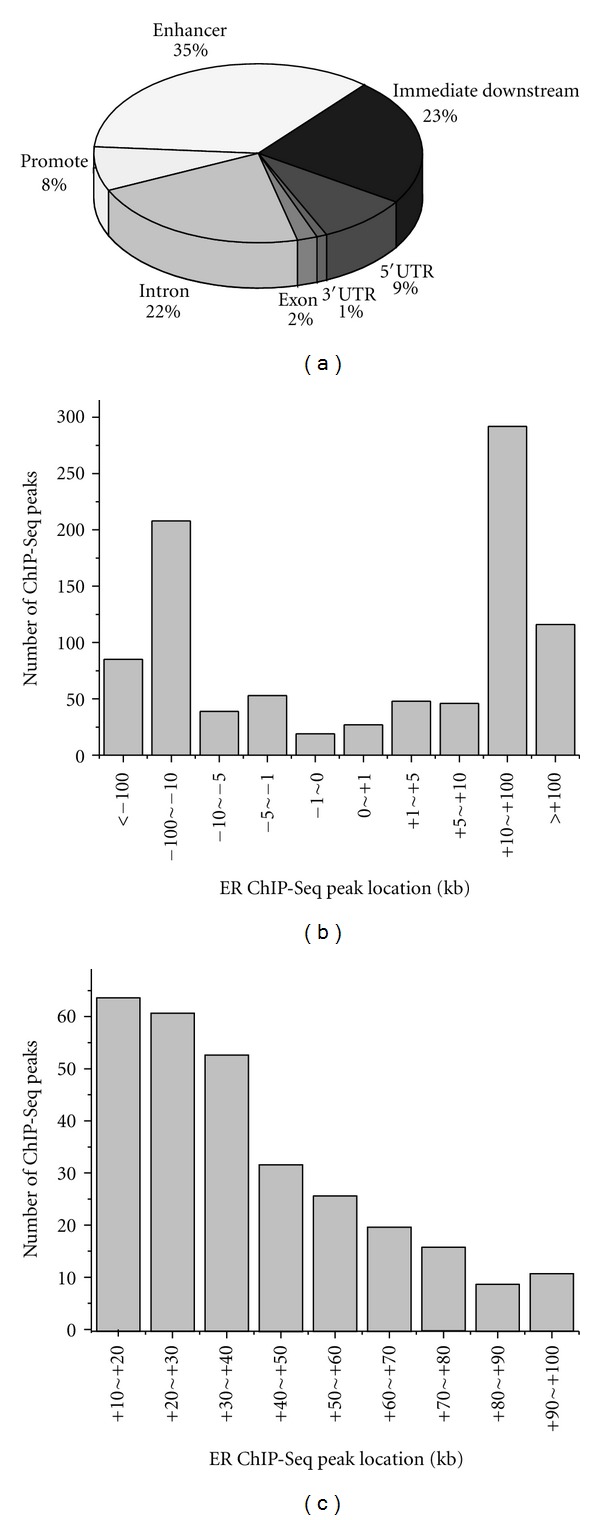
Location analysis of ER binding sites. (a) locations relative to nearest-neighbor genes. (b) Genomic Locations of ER ChIP-Seq peaks. (c) Genomic locations of ER ChIP-Seq peaks within +10~+100 kb.

Furthermore, we examined the location of ER enrichment sites relativer to the nearest-neighbor genes. The result was shown in Figure 4(a). Only 8% (72) of the peaks occured within gene promoters (defined here as within 5 kb upstream of 5′ to TSS). Also, 34% (317) of the peaks resided in intragenic sites, including 1% (10) in the 3′UTR, 9% (81) in the 5′UTR, 2% (20) in the exon, and 22% (206) in the intron. The occupancy of enhancer (>5 kb away 5′ to TSS) was 35% (332). According to Figure 4(b), the peaks occurred most frequently between −10 kb to −100 kb, +10 kb to +100 kb, with +10 kb to +100 kb being the highest. A further insight into the peaks within +10 kb to +100 kb showed that peaks were preferably located within the regions spanning from +10 kb to +40 kb (Figure 4(c)).

### 3.2. Using Gene Expression Data to Confirm the ER Binding Sites

In order to determine the specific gene responses corresponding to ER in MCF-7 cells, we compared the nearest-neighbor genes of ER binding sites to the published studies examining differentially expressed genes between ER+ and ER− breast tumors. We used the 3 studies in Table 4 for the gene expression analysis. Differentially expressed genes were selected based on a *q*-value cut-off of less than 1% using a stringent statistical analysis method. We identified 5692 and 6101 up- and downregulated genes. When combined with the nearest-neighbor genes of ER binding sites, 289 up-regulated genes and 198 down-regulated genes were associated with the ER binding sites (see additional file 1, Supplementary Material available online at doi:10.1155/2012/568950). Among these genes, 33 upregulated genes and 11 downregulated genes were also identified by published ChIP-PET analysis [27].

**Figure 5:**
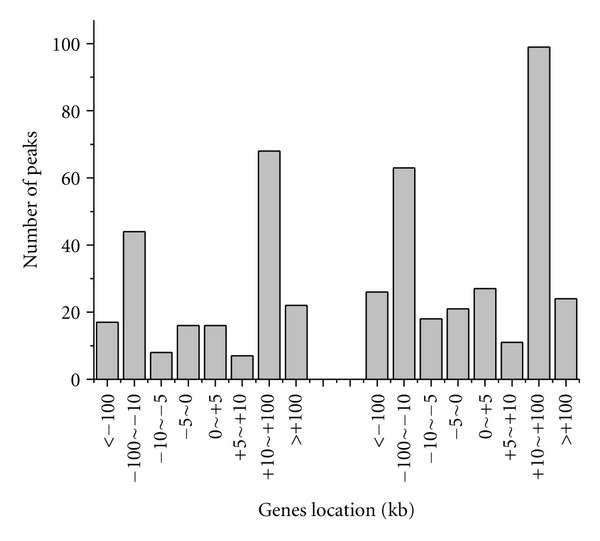
Genomic Locations of differentially expressed genes in the vicinity of ER binding sites.

Our analysis found that more binding sites were associated with ER up-regulated genes (60%) compared to down-regulated genes (40%), indicating that ER was more frequently involved in the direct regulation of up-regulated genes. We also examined the location of ER binding sites in up-regulated and down-regulated genes. As shown in Figure 5, both the up- and down-regulated genes occurred most frequently between −10 kb to −100 kb, +10 kb to +100 kb, which verified the long-range control mode of ER factor.

### 3.3. SNPs Occurred near the ER Binding Sites

Current studies have shown that the breast cancer risks are associated with commonly occurring single nucleotide polymorphisms (SNPs) [28–32]. The table SNP (131) (dbSNP build 131) in UCSC (http://genome.ucsc.edu/) was used to identify SNPs near the ER binding sites. A total of 2694 SNP loci were found and subsequently annotated using dbSNP in NCBI.

Compared with the differently expressed gene set in the vicinity of ER binding sites, 836 SNPs in or near 157 ER-regulated genes were identified (see additional file 2). Most of the SNPs (94.5%) were located in intron and untranslated regions. Only 5.5% were located in the regions of near-gene, coding-synon, missense, and frameshift. These SNPs might have close relationship with breast cancer.

### 3.4. Functional Annotation of ER Binding Sites

To identify the biological processes and pathways altered by ER, we employed three functional annotation systems, the Gene Ontology (GO) categories [20], canonical KEGG Pathway Maps [21], and commercial software MetaCore-GeneGo Pathway Maps, to perform the enrichment analysis for gene function. 

**Figure 6:**
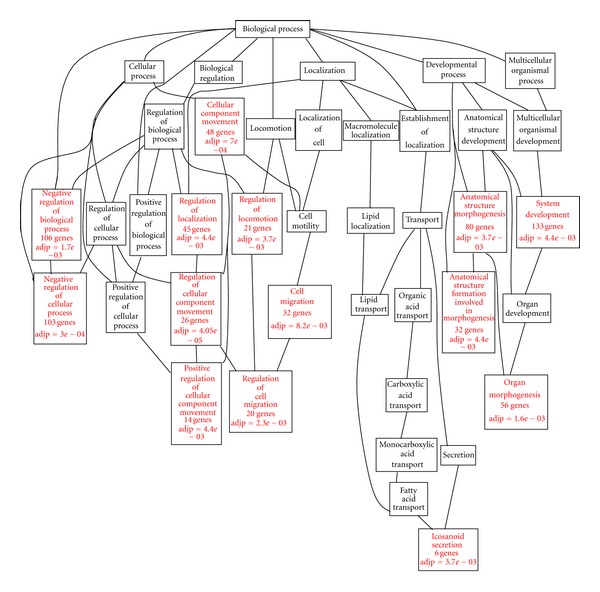
Directed Acyclic Graphs (DAGs) of significantly enriched GO (Gene Ontology) categories (*P* < 0.01).

To gain an overview of the biological processes in which the nearest-neighbor genes of ER binding sites reside, we firstly performed gene set enrichment analysis using Gene Ontology database. Statistically significant (Hypergeometric test, *P*-value < 0.01) enriched GO terms were identified using the web tool GOTM (Gene Ontology Tree Machine) [22]. The Gene Ontology Directed Acyclic Graph for the nearest-neighbor genes generated by GOTM was presented in Figure 6. The terms with red color were significantly enriched. In terms of biological process, negative regulation of biological process and cellular process, cellular component movement, and regulation of localization and locomotion, structure and system development were significantly enriched. Furthermore, whether differently expressed or not, genes were mostly associated with biological regulation and metabolic process in biological process terms, protein binding in molecular function terms, and membrane in cellular component terms (each term included more than 100 genes). Gene functions for all the nearest-neighbor genes were summarized in Table 5.

The KEGG Pathway database (posted on May 23, 2011) was used to identify functional modules regulated by ER. Seventeen significantly enriched pathways (*P*-value < 0.01) were revealed (Table 6). In these pathways, most genes were also differentially expressed between ER+ and ER− tumors. Pathways in cancer, focal adhesion, axon guidance, regulation of actin cytoskeleton, and MAPK signaling pathway ranked among the most enriched pathways. The top enriched maps, such as focal adhesion pathway and MAPK signaling pathway, were reported to be related with ER in breast cancer. High expression of focal adhesion kinase had been reported to be related to cancer progression of breast. And tumors with high expression of focal adhesion kinase lack ER and PR [33]. It was also reported that hyperactivation of MAPK could repress the ER expression in breast tumors [34]. Pathways in cancer were the top enriched KEGG pathway. The abnormal expression of some genes occurred in several types of cancer [35–37]. Axon guidance pathway played important roles in cancers. Axon guidance molecules might control the development, migration, and invasion of cancer cells [38]. Regulation of actin cytoskeleton was related to cancer cell migration and invasion [39]. This indicated the crucial role of ER in the development, migration, and invasion of breast cancer. 

GeneGo was also used to perform the pathway analysis. Ten pathways were found to be significantly enriched with *P*-value < 0.01 and FDR < 0.05 (Table 7). The result showed that ER binding sites were enriched in breast cancer related pathways. Among the top five maps, development_prolactin receptor signaling and development_glucocorticoid receptor signaling had been reported to associate with ER [40, 41]. development_ligand-independent activation of ESR1 and ESR2 was another enriched map which might have close relationship with ER. APRIL and BAFF were the members of tumor necrosis factor family which related to a plethora of cellular events from proliferation and differentiation to apoptosis and tumor reduction [42]. IL-22 might play a role in the control of tumor growth and progression in breast [43]. However, the relationship between ER and these two pathways need further experimental study.

## 4. Conclusions

ER is an important molecular symbol of breast cancer. A full understanding of the molecular mechanisms of ER will be useful for the research in the prediction and treatment of breast cancer. The ChIP-Seq technology is useful to study the interaction of protein and DNA on a genome-wide scale. ChIP-Seq data can effectively analyze the regulatory mechanism of transcription factor in genome-wide scale. In this study, we used ChIP-Seq data to identify the global sites regulated by ER in MCF-7 breast cancer cell line. In order to get more reliable result, three different tools were used to analyze two datasets. And 933 binding sites were identified, and the ERE motif was refined here.

The analysis of the global genomic occupancy of ER-regulated genes revealed that 92% of the total 933 ER-binding sites were located far away from promoters. This suggested that the canonical mode of ER factor function involved long-range control. Previous research had reported that ER-*α* includes looping [44]. Using ChIP-PET, Lin et al. [27] had analyzed the genome-wide ER-*α* chromatin occupancy and revealed abundant nonpromoter sites. Our findings provided further support for this mode of ER factor function.

We compared the ER binding sites found in this study with published differentially expressed genes between ER+ and ER− breast tumors. A set of 487 genes was found significant in discriminating ER status in breast tumors. This indicated that these genes appeared to affect ER response. Only 9% (44) of the genes have been identified by Lin et al. [27], while the remaining need further validations. We found that binding sites were preferentially associated with ER up-regulated genes, indicating that ER was more frequently involved in the regulation of upregulated genes. The location of 487 genes verified the long-range control mode of ER factor.

In this study, we found 2694 single nucleotide polymorphisms loci located in or near the ER binding sites. Among these SNPs, the 157 genes of 836 SNPs were also differentially expressed between ER+ and ER− breast tumors. It indicated that this set of SNPs might have close relationship with ER in breast. 

The functional annotation provided a deeper understanding of ER and ER-associated genes. Enrichment analysis of GO gave an overview of gene function. As shown in Figure 6, significantly enriched terms belonged to three classes, biological regulation, cellular processes, and developmental processes. The result of KEGG enrichment analysis was similar. Five pathways were involved in cellular processes, including focal adhesion, regulation of actin cytoskeleton, oocyte meiosis, endocytosis, and p53 signaling pathway. These pathways were associated with cell communication, movement, growth, and death. Most enriched terms determined by GeneGO were development pathways. It was suggested that ER-regulated genes participated in various development processes. Moreover, KEGG pathway analysis suggested that ER-regulated genes were enriched in some diseases related pathways. Both KEGG and GeneGO pathway analysis revealed that some immune-related pathways were enriched, such as chemokine signaling pathway and immune response_IL-22 signaling pathway. These results indicated that ER-regulated genes related to the development, progression, and metastasis of breast. ER affected every developed stage of breast. However, the regulatory mechanisms of ER in different stages and different pathways still need further studies.

## Supplementary Material

Additional file 1 shows the differentially expressed genes in the vicinity of ER binding sites. Using 3 gene expression studies and a criterion of q-value <0.01, we identified 5692 and 6101 up- and down-regulated down-regulated genes between ER+ and ER- breast tumors. Among these genes, 289 up-regulated and 198 down-regulated genes located near the ER binding sites. The details of these 489 estrogen response elements were listed here. Additional file 2 shows the SNPs occurred in the ER-regulated genes located near the binding sites. These SNPs were identified by using the table SNP (131) (dbSNP build 131) in UCSC (http://genome.ucsc.edu/). Totally, 836 SNPs in or near 157 ER-regulated genes were identified.

## Figures and Tables

**Table 1:** The CHIP-Seq datasets.

Dataset	Platform	Cell line	Sample information
GSE19013	Illumina	MCF-7	Ethanol treated
			E2-treated
GSE14664	Illumina	MCF-7	ER_minus_ligand
			ER_E2

**Table 2:** An overview of the characteristics of different Chip-Seq peak detection algorithm.

Algorithm	Profile	Background model	Control sample	Use control to compute FDR
F-Seq	Kernel density estimation (KDE)		√	
FindPeaks	Aggregation of overlapped tags	Monte Carlo		
SISSRs	Window scan	Poisson	√	
QuEST	Kernel density estimation (KDE)		√	√
MACS	Tags shifted then window scan	dynamic Poisson	√	√
CisGenome	Strand-specific window scan	Negative binomial	√	√

**Table 3:** Number of ER binding sites identified by three ChIP-Seq peak calling programs (FDR < 0.01).

	Number of ER binding sites				
Dataset				Number of overlaped sites	
	CisGenome	MACS	QuEST		
GSE19013	8137	5583	5418	2019	933
GSE14664	6773	7765	9280	5061	

**Table 4:** Breast cancer gene expression dataset and differently expressed genes number (*q*-value < 1%).

Author	Journal	Array type	Sample *N* ER+	Sample *N* ER−	Differently expressed genes	
					Upregulated	Downregulated
Graham et al. [24]	Clin Cancer Res	Affy	15	15	709	333
Wang et al. [25]	Lancet	Affy	209	77	2081	2537
Lu et al. [26]	Breast Cancer Res Treat	Affy	76	53	5136	5445

All					5692	6101

**Table 5:** The comparison of top enriched GO categories between different expressed and other nearest-neighbor genes of ER binding sites (number of genes ≥ 100).

Genes set	Biological process	Molecular function	Cellular component
Differently expressed	Biological regulation, metabolic process, cell communication, organismal process, localization, developmental process	Protein binding, iron binding	Membrane, nucleus
Others	Biological regulation, metabolic process	Protein binding	Membrane

**Table 6:** KEGG pathways enriched with the nearest-neighbor genes of ER binding sites (*P*-value < 0.01).

KEGG ID	Pathways name	*P*-value	Number of genes	Number of different expressed genes
hsa05200	Pathways in cancer	2.24*E* − 05	22	16
hsa04510	Focal adhesion	0.0002	15	14
hsa04360	Axon guidance	0.0009	11	8
hsa04810	Regulation of actin cytoskeleton	0.0012	14	11
hsa04010	MAPK signaling pathway	0.0022	15	12
hsa04114	Oocyte meiosis	0.0024	9	8
hsa04144	Endocytosis	0.0024	12	11
hsa04115	p53 signaling pathway	0.0024	7	7
hsa05216	Thyroid cancer	0.0024	5	4
hsa05218	Melanoma	0.0033	7	3
hsa04020	Calcium signaling pathway	0.004	11	4
hsa04062	Chemokine signaling pathway	0.0064	11	9
hsa04914	Progesterone-mediated oocyte maturation	0.0085	7	7
hsa01100	Metabolic pathways	0.0086	35	28
hsa00450	Selenoamino acid metabolism	0.0088	4	3
hsa05414	Dilated cardiomyopathy	0.0096	7	7
hsa03440	Homologous recombination	0.0097	4	3

**Table 7:** Terms of the enriched GeneGo pathway maps (*P*-value < 0.01, FDR < 0.05).

GeneGo pathway terms	*P*-value
Apoptosis and survival_APRIL and BAFF signaling	1.29889*E* − 05
Development_prolactin receptor signaling	4.95517*E* − 05
Development_glucocorticoid receptor signaling	5.81237*E* − 05
Development_ligand-independent activation of ESR1 and ESR2	0.000295251
Immune response_IL-22 signaling pathway	0.000381484
Development_EPO-induced Jak-STAT pathway	0.000531744
Development_growth hormone signaling via STATs and PLC/IP3	0.000531744
Cytoskeleton remodeling_keratin filaments	0.000622315
Development_GM-CSF signaling	0.000660576
Transcription_transcription regulation of aminoacid metabolism	0.000752764
