# Supplementary material for: Identification and Functional Annotation of Genome-Wide ER-Regulated Genes in Breast Cancer Based on ChIP-Seq Data
Source: Comput Math Methods Med. 2012 Dec 31;2012:568950. doi: 10.1155/2012/568950 (PMC3546463; doi:10.1155/2012/568950)
Supplement: Supplementary file 1 — Additional file 1 shows the differentially expressed genes in the vicinity of ER binding sites. Using 3 gene expression studies and a criterion of q-value <0.01, we identified 5692 and 6101 up- and down-regulated down-regulated genes between ER+ and ER- breast tumors. Among these genes, 289 up-regulated and 198 down-regulated genes located near the ER binding sites. The details of these 489 estrogen response elements were listed here. Additional file 2 shows the SNPs occurred in the ER-regulated genes located near the binding sites. These SNPs were identified by using the table SNP (131) (dbSNP build 131) in UCSC (http://genome.ucsc.edu/). Totally, 836 SNPs in or near 157 ER-regulated genes were identified. [file 568950.f1.pdf]

Additional file 1 – Differentially expressed genes in the vicinity of ER binding sites

RefSeq_ID	Gene Symbol	Chromosome	Expression Level	
			Up-regulated	Down-regulated
NR_028386	LOC375196	chr2		+
NR_027322	LOC283070	chr10		+
NR_026955	LOC121838	chr13		+
NR_026943	LOC642852	chr21		+
NR_026800	KIAA0125	chr14		+
NR_026773	C6orf123	chr6		+
NR_024576	SEC61A2	chr10		+
NM_199005	ZNF322B	chr9		+
NM_197956	NAIF1	chr9		+
NM_183419	RNF19A	chr8		+
NM_182757	RNF144B	chr6		+
NM_181709	FAM101A	chr12		+
NM_181671	PITPNC1	chr17		+
NM_181354	OXR1	chr8		+
NM_181054	HIF1A	chr14		+
NM_178815	ARL5B	chr10		+
NM_173515	CNKSR3	chr6		+
NM_173354	SIK1	chr21		+
NM_153229	TMEM92	chr17		+
NM_152903	KBTBD6	chr13		+
NM_152830	ACE	chr17		+
NM_152365	C1orf172	chr1		+
NM_152308	C16orf75	chr16		+
NM_152295	TARS	chr5		+
NM_152243	CDC42EP1	chr22		+
NM_147184	TP53I3	chr2		+
NM_145804	ABTB2	chr11		+
NM_144665	SESN3	chr11		+
NM_139283	PPTC7	chr12		+
NM_138572	TAF8	chr6		+
NM_133510	RAD51L1	chr14		+
NM_130445	COL18A1	chr21		+
NM_080927	DCBLD2	chr3		+
NM_053031	MYLK	chr3		+
NM_052929	FHAD1	chr1		+
NM_052870	SNX18	chr5		+
NM_052864	TIFA	chr4		+
NM_033508	GCK	chr7		+
NM_033379	CDC2	chr10		+

NM_033069	C6orf114	chr6		+
NM_033023	PDGFA	chr7		+
NM_032873	UBASH3B	chr11		+
NM_032717	AGPAT9	chr4		+
NM_032211	LOXL4	chr10		+
NM_032148	SLC41A2	chr12		+
NM_032048	EMILIN2	chr18		+
NM_031965	GSG2	chr17		+
NM_031892	SH3KBP1	chrX		+
NM_030797	FAM49A	chr2		+
NM_024605	ARHGAP10	chr4		+
NM_024587	TMEM53	chr1		+
NM_024524	ATP13A3	chr3		+
NM_024076	KCTD15	chr19		+
NM_024070	PVRIG	chr7		+
NM_021177	LSM2	chr6		+
NM_020947	KIAA1609	chr16		+
NM_020845	PITPNM2	chr12		+
NM_020805	KLHL14	chr18		+
NM_020803	KLHL8	chr4		+
NM_020458	TTC7A	chr2		+
NM_020397	CAMK1D	chr10		+
NM_020382	SETD8	chr12		+
NM_020225	STOX2	chr4		+
NM_018990	SASH3	chrX		+
NM_018697	LANCL2	chr7		+
NM_018649	H2AFY2	chr10		+
NM_018641	CHST12	chr7		+
NM_018482	ASAP1	chr8		+
NM_018265	C1orf106	chr1		+
NM_018112	TMEM38B	chr9		+
NM_018006	TRMU	chr22		+
NM_017791	FLVCR2	chr14		+
NM_017784	OSBPL10	chr3		+
NM_017628	TET2	chr4		+
NM_017596	KIF21B	chr1		+
NM_017551	GRID1	chr10		+
NM_017519	ARID1B	chr6		+
NM_017490	MARK2	chr11		+
NM_017436	A4GALT	chr22		+
NM_016448	DTL	chr1		+
NM_016445	PLEK2	chr14		+
NM_016282	AK3	chr9		+

NM_016071	MRPS33	chr7		+
NM_016058	TPRKB	chr2		+
NM_015991	C1QA	chr1		+
NM_015379	BRI3	chr7		+
NM_015259	ICOSLG	chr21		+
NM_015215	CAMTA1	chr1		+
NM_015112	MAST2	chr1		+
NM_015093	MAP3K7IP2	chr6		+
NM_015029	POP1	chr8		+
NM_015018	DOPEY1	chr6		+
NM_014732	KIAA0513	chr16		+
NM_014696	GPRIN2	chr10		+
NM_014553	TFCP2L1	chr2		+
NM_014363	SACS	chr13		+
NM_014214	IMPA2	chr18		+
NM_014176	UBE2T	chr1		+
NM_013336	SEC61A1	chr3		+
NM_012293	PXDN	chr2		+
NM_012259	HEY2	chr6		+
NM_012100	DNPEP	chr2		+
NM_012083	FRAT2	chr10		+
NM_007355	HSP90AB1	chr6		+
NM_007107	SSR3	chr3		+
NM_007080	LSM6	chr4		+
NM_007024	TMEM115	chr3		+
NM_006941	SOX10	chr22		+
NM_006808	SEC61B	chr9		+
NM_006795	EHD1	chr11		+
NM_006607	PTTG2	chr4		+
NM_006482	DYRK2	chr12		+
NM_006203	PDE4D	chr5		+
NM_006080	SEMA3A	chr7		+
NM_005792	MPHOSPH6	chr16		+
NM_005729	PPIF	chr10		+
NM_005720	ARPC1B	chr7		+
NM_005596	NFIB	chr9		+
NM_005582	CD180	chr5		+
NM_005434	MALL	chr2		+
NM_005402	RALA	chr7		+
NM_005360	MAF	chr16		+
NM_005297	MCHR1	chr22		+
NM_005253	FOSL2	chr2		+
NM_004972	JAK2	chr9		+

NM_004952	EFNA3	chr1		+
NM_004930	CAPZB	chr1		+
NM_004924	ACTN4	chr19		+
NM_004782	SNAP29	chr22		+
NM_004745	DLGAP2	chr8		+
NM_004690	LATS1	chr6		+
NM_004669	CLIC3	chr9		+
NM_004595	SMS	chrX		+
NM_004586	RPS6KA3	chrX		+
NM_004578	RAB4A	chr1		+
NM_004394	DAP	chr5		+
NM_004387	NKX2-5	chr5		+
NM_004215	EBAG9	chr8		+
NM_003954	MAP3K14	chr17		+
NM_003935	TOP3B	chr22		+
NM_003770	KRT37	chr17		+
NM_003745	SOCS1	chr16		+
NM_003576	STK24	chr13		+
NM_003488	AKAP1	chr17		+
NM_003374	VDAC1	chr5		+
NM_003222	TFAP2C	chr20		+
NM_002980	SCTR	chr2		+
NM_002568	PABPC1	chr8		+
NM_002547	OPHN1	chrX		+
NM_002485	NBN	chr8		+
NM_002467	MYC	chr8		+
NM_002271	IPO5	chr13		+
NM_002163	IRF8	chr16		+
NM_002144	HOXB1	chr17		+
NM_002061	GCLM	chr1		+
NM_002046	GAPDH	chr12		+
NM_001987	ETV6	chr12		+
NM_001962	EFNA5	chr5		+
NM_001935	DPP4	chr2		+
NM_001905	CTPS	chr1		+
NM_001874	CPM	chr12		+
NM_001838	CCR7	chr17		+
NM_001453	FOXC1	chr6		+
NM_001427	EN2	chr7		+
NM_001163817	DHCR7	chr11		+
NM_001161442	SH2D2A	chr1		+
NM_001161440	PTPRH	chr19		+
NM_001144033	STOML3	chr13		+

NM_001142864	FAM38A	chr16		+
NM_001142776	CHAC1	chr15		+
NM_001135242	NDRG1	chr8		+
NM_001134479	LRRC8D	chr1		+
NM_001130716	PLAC8	chr4		+
NM_001130455	DYSF	chr2		+
NM_001130141	PCBP3	chr21		+
NM_001126121	SLC25A19	chr17		+
NM_001102	ACTN1	chr14		+
NM_001099856	IKBKG	chrX		+
NM_001099786	ICAM2	chr17		+
NM_001099285	PTMA	chr2		+
NM_001098815	KIAA0748	chr12		+
NM_001098270	MGAT3	chr22		+
NM_001082959	SCARB1	chr12		+
NM_001040446	MTMR12	chr5		+
NM_001013436	MPST	chr22		+
NM_001005389	NFASC	chr1		+
NM_001005190	OR7A10	chr19		+
NM_001004416	UMODL1	chr21		+
NM_001003395	TPD52L1	chr6		+
NM_001001740	RFWD2	chr1		+
NM_001001392	CD44	chr11		+
NM_000977	RPL13	chr16		+
NM_000944	PPP3CA	chr4		+
NM_000701	ATP1A1	chr1		+
NM_000700	ANXA1	chr9		+
NM_000573	CR1	chr1		+
NM_000274	OAT	chr10		+
NM_000189	HK2	chr2		+
NR_030691	ANO1	chr11	+	
NR_028408	LOC400027	chr12	+	
NR_028048	CRAT	chr9	+	
NR_027868	PDCD6IP	chr3	+	
NR_026779	C14orf139	chr14	+	
NR_026590	CDYL	chr6	+	
NR_024555	RHOBTB1	chr10	+	
NR_003587	MYO15B	chr17	+	
NR_003367	PVT1	chr8	+	
NM_213613	SLC26A1	chr4	+	
NM_213601	TMED8	chr14	+	
NM_207446	FAM174B	chr15	+	
NM_206967	C16orf74	chr16	+	

NM_203458	NOTCH2NL	chr1	+	
NM_203284	RBPJ	chr4	+	
NM_201557	FHL2	chr2	+	
NM_198514	NHLRC2	chr10	+	
NM_198461	LONRF2	chr2	+	
NM_194278	C14orf43	chr14	+	
NM_183357	ADCY5	chr3	+	
NM_182920	ADAMTS9	chr3	+	
NM_181784	SPRED2	chr2	+	
NM_181481	C18orf1	chr18	+	
NM_178126	FAM134C	chr17	+	
NM_177964	LYPD6B	chr2	+	
NM_175931	CBFA2T3	chr16	+	
NM_175058	PLEKHA7	chr11	+	
NM_174886	TGIF1	chr18	+	
NM_173527	REM2	chr14	+	
NM_173505	ANKRD29	chr18	+	
NM_170721	MSI2	chr17	+	
NM_153812	PHF13	chr1	+	
NM_153322	PMP22	chr17	+	
NM_152900	MAGI3	chr1	+	
NM_152793	C7orf41	chr7	+	
NM_152573	RASEF	chr9	+	
NM_152400	C4orf32	chr4	+	
NM_152396	METTL6	chr3	+	
NM_152263	TPM3	chr1	+	
NM_152231	FBXO34	chr14	+	
NM_145253	FAM100A	chr16	+	
NM_144973	DENND5B	chr12	+	
NM_144628	TBC1D20	chr20	+	
NM_138634	MSMB	chr10	+	
NM_138473	SP1	chr12	+	
NM_133373	PLCD3	chr17	+	
NM_080391	PTP4A2	chr1	+	
NM_053056	CCND1	chr11	+	
NM_033127	SEC16B	chr1	+	
NM_032199	ARID5B	chr10	+	
NM_032104	PPP1R12B	chr1	+	
NM_030793	FBXO38	chr5	+	
NM_030762	BHLHE41	chr12	+	
NM_025268	TMEM121	chr14	+	
NM_025191	EDEM3	chr1	+	
NM_025134	CHD9	chr16	+	

NM_024979	MCF2L	chr13	+	
NM_024815	NUDT18	chr8	+	
NM_024607	PPP1R3B	chr8	+	
NM_024336	IRX3	chr16	+	
NM_024071	ZFYVE21	chr14	+	
NM_023037	FRY	chr13	+	
NM_022748	TNS3	chr7	+	
NM_022648	TNS1	chr2	+	
NM_022467	CHST8	chr19	+	
NM_022358	KCNK15	chr20	+	
NM_022151	MOAP1	chr14	+	
NM_022132	MCCC2	chr5	+	
NM_022068	FAM38B	chr18	+	
NM_021800	DNAJC12	chr10	+	
NM_021215	RPRD1B	chr20	+	
NM_021183	RAP2C	chrX	+	
NM_021168	RAB40C	chr16	+	
NM_020859	SHROOM3	chr4	+	
NM_020820	PREX1	chr20	+	
NM_020775	KIAA1324	chr1	+	
NM_020689	SLC24A3	chr20	+	
NM_020531	C20orf3	chr20	+	
NM_020440	PTGFRN	chr1	+	
NM_020424	LYRM1	chr16	+	
NM_020244	CHPT1	chr12	+	
NM_020205	OTUD7B	chr1	+	
NM_020190	OLFML3	chr1	+	
NM_020145	SH3GLB2	chr9	+	
NM_019020	TBC1D16	chr17	+	
NM_018976	SLC38A2	chr12	+	
NM_018344	SLC29A3	chr10	+	
NM_018271	THNSL2	chr2	+	
NM_017782	C10orf18	chr10	+	
NM_017771	PXK	chr3	+	
NM_017770	ELOVL2	chr6	+	
NM_017583	TRIM44	chr11	+	
NM_017423	GALNT7	chr4	+	
NM_016463	CXXC5	chr5	+	
NM_016441	CRIM1	chr2	+	
NM_016346	NR2E3	chr15	+	
NM_016121	KCTD3	chr1	+	
NM_016048	ISOC1	chr5	+	
NM_015886	PI15	chr8	+	

NM_015635	GAPVD1	chr9	+	
NM_015608	C10orf137	chr10	+	
NM_015595	SGEF	chr3	+	
NM_015559	SETBP1	chr18	+	
NM_015525	IBTK	chr6	+	
NM_015522	DYNC2LI1	chr2	+	
NM_015404	DFNB31	chr9	+	
NM_015335	MED13L	chr12	+	
NM_015317	PUM2	chr2	+	
NM_015221	DNMBP	chr10	+	
NM_015168	ZC3H4	chr19	+	
NM_015130	TBC1D9	chr4	+	
NM_015111	N4BP3	chr5	+	
NM_014992	DAAM1	chr14	+	
NM_014945	ABLIM3	chr5	+	
NM_014899	RHOBTB3	chr5	+	
NM_014873	LPGAT1	chr1	+	
NM_014779	TSC22D2	chr3	+	
NM_014668	GREB1	chr2	+	
NM_014633	CTR9	chr11	+	
NM_014583	LMCD1	chr3	+	
NM_014488	RAB30	chr11	+	
NM_014365	HSPB8	chr12	+	
NM_014267	C11orf58	chr11	+	
NM_014246	CELSR1	chr22	+	
NM_013262	MYLIP	chr6	+	
NM_013240	N6AMT1	chr21	+	
NM_012398	PIP5K1C	chr19	+	
NM_012330	MYST4	chr10	+	
NM_012318	LETM1	chr4	+	
NM_012279	ZNF346	chr5	+	
NM_007218	RNF139	chr8	+	
NM_007173	PRSS23	chr11	+	
NM_007106	UBL3	chr13	+	
NM_007011	ABHD2	chr15	+	
NM_007008	RTN4	chr2	+	
NM_006885	ZFHX3	chr16	+	
NM_006868	RAB31	chr18	+	
NM_006788	RALBP1	chr18	+	
NM_006710	COPS8	chr2	+	
NM_006645	STARD10	chr11	+	
NM_006633	IQGAP2	chr5	+	
NM_006621	AHCYL1	chr1	+	

NM_006556	PMVK	chr1	+	
NM_006513	SARS	chr1	+	
NM_006379	SEMA3C	chr7	+	
NM_006327	TIMM23	chr10	+	
NM_006148	LASP1	chr17	+	
NM_006108	SPON1	chr11	+	
NM_006094	DLC1	chr8	+	
NM_006019	TCIRG1	chr11	+	
NM_006009	TUBA1A	chr12	+	
NM_005794	DHRS2	chr14	+	
NM_005781	TNK2	chr3	+	
NM_005749	TOB1	chr17	+	
NM_005742	PDIA6	chr2	+	
NM_005723	TSPAN5	chr4	+	
NM_005578	LPP	chr3	+	
NM_005392	PHF2	chr9	+	
NM_005375	MYB	chr6	+	
NM_005252	FOS	chr14	+	
NM_005128	DOPEY2	chr21	+	
NM_005050	ABCD4	chr14	+	
NM_004926	ZFP36L1	chr14	+	
NM_004747	DLG5	chr10	+	
NM_004725	BUB3	chr10	+	
NM_004531	MOCS2	chr5	+	
NM_004491	GRLF1	chr19	+	
NM_004465	FGF10	chr5	+	
NM_004430	EGR3	chr8	+	
NM_004310	RHOH	chr4	+	
NM_004281	BAG3	chr10	+	
NM_004235	KLF4	chr9	+	
NM_004171	SLC1A2	chr11	+	
NM_004104	FASN	chr17	+	
NM_004078	CSRP1	chr1	+	
NM_004041	ARRB1	chr11	+	
NM_004040	RHOB	chr2	+	
NM_003898	SYNJ2	chr6	+	
NM_003848	SUCLG2	chr3	+	
NM_003714	STC2	chr5	+	
NM_003613	CILP	chr15	+	
NM_003568	ANXA9	chr1	+	
NM_003565	ULK1	chr12	+	
NM_003489	NRIP1	chr21	+	
NM_003474	ADAM12	chr10	+	

NM_003463	PTP4A1	chr6	+	
NM_003439	ZKSCAN1	chr7	+	
NM_003358	UGCG	chr9	+	
NM_003300	TRAF3	chr14	+	
NM_003246	THBS1	chr15	+	
NM_003226	TFF3	chr21	+	
NM_003225	TFF1	chr21	+	
NM_003134	SRP14	chr15	+	
NM_003060	SLC22A5	chr5	+	
NM_002957	RXRA	chr9	+	
NM_002911	UPF1	chr19	+	
NM_002841	PTPRG	chr3	+	
NM_002665	PLGLB2	chr2	+	
NM_002583	PAWR	chr12	+	
NM_002449	MSX2	chr5	+	
NM_002430	MN1	chr22	+	
NM_002413	MGST2	chr4	+	
NM_002412	MGMT	chr10	+	
NM_002273	KRT8	chr12	+	
NM_002213	ITGB5	chr3	+	
NM_002154	HSPA4	chr5	+	
NM_002111	HTT	chr4	+	
NM_002074	GNB1	chr1	+	
NM_002052	GATA4	chr8	+	
NM_002019	FLT1	chr13	+	
NM_002014	FKBP4	chr12	+	
NM_001795	CDH5	chr16	+	
NM_001769	CD9	chr12	+	
NM_001756	SERPINA6	chr14	+	
NM_001706	BCL6	chr3	+	
NM_001632	ALPP	chr2	+	
NM_001584	MPPED2	chr11	+	
NM_001552	IGFBP4	chr17	+	
NM_001497	B4GALT1	chr9	+	
NM_001408	CELSR2	chr1	+	
NM_001329	CTBP2	chr10	+	
NM_001310	CREBL2	chr12	+	
NM_001218	CA12	chr15	+	
NM_001166294	SSX2IP	chr1	+	
NM_001166263	MTIF3	chr13	+	
NM_001166108	PALLD	chr4	+	
NM_001164595	PDZRN4	chr12	+	
NM_001164317	FLNB	chr3	+	

NM_001163260	FAM63A	chr1	+	
NM_001161841	SULF2	chr20	+	
NM_001161661	WWC1	chr5	+	
NM_001159770	SLC39A11	chr17	+	
NM_001159694	EMCN	chr4	+	
NM_001145443	PFKFB3	chr10	+	
NM_001144758	PHLDB1	chr11	+	
NM_001143989	NBPF4	chr1	+	
NM_001143962	CAPN8	chr1	+	
NM_001143819	TPCN1	chr12	+	
NM_001142272	RAB11FIP3	chr16	+	
NM_001134473	KIAA0182	chr16	+	
NM_001134368	SLC6A6	chr3	+	
NM_001130958	FABP6	chr5	+	
NM_001127714	HIVEP3	chr1	+	
NM_001122742	ESR1	chr6	+	
NM_001122606	LAMP2	chrX	+	
NM_001116	ADCY9	chr16	+	
NM_001113239	HIPK2	chr7	+	
NM_001105568	KIF13A	chr6	+	
NM_001105192	TLE3	chr15	+	
NM_001100913	PACS2	chr14	+	
NM_001100590	KIAA0232	chr4	+	
NM_001081562	DMPK	chr19	+	
NM_001080479	RGNEF	chr5	+	
NM_001080414	CCDC88C	chr14	+	
NM_001079539	XBP1	chr22	+	
NM_001077484	SLC38A1	chr12	+	
NM_001077397	IRF2BP2	chr1	+	
NM_001042665	PLEKHG5	chr1	+	
NM_001042353	FAM110A	chr20	+	
NM_001039360	ZBTB7C	chr18	+	
NM_001035223	RGL3	chr19	+	
NM_001034850	FAM134B	chr5	+	
NM_001033057	MAGI1	chr3	+	
NM_001032391	LCMT1	chr16	+	
NM_001029851	PDE8B	chr5	+	
NM_001024956	SC5DL	chr11	+	
NM_001024074	HNMT	chr2	+	
NM_001018074	NR3C1	chr5	+	
NM_001014797	KCNMA1	chr10	+	
NM_001006657	WDR35	chr2	+	
NM_001005914	SEMA3B	chr3	+	

NM_001005476	PKP4	chr2	+	
NM_001005159	SFMBT1	chr3	+	
NM_001003698	RREB1	chr6	+	
NM_001002914	KCTD11	chr17	+	
NM_001002295	GATA3	chr10	+	
NM_001001664	SPOPL	chr2	+	
NM_000926	PGR	chr11	+	
NM_000720	CACNA1D	chr3	+	
NM_000633	BCL2	chr18	+	
NM_000623	BDKRB2	chr14	+	
NM_000618	IGF1	chr12	+	
NM_000609	CXCL12	chr10	+	
NM_000552	VWF	chr12	+	
NM_000501	ELN	chr7	+	
NM_000366	TPM1	chr15	+	
NM_000283	PDE6B	chr4	+	
NM_000224	KRT18	chr12	+	
NM_000153	GALC	chr14	+	

Additional file 2 –Candidate SNPs

**Chromosome 1**
ANXA9:rs78982670,rs607518,rs3754210
CAMTA1:rs11120798,rs80085554,rs78547373,rs35190009,rs1013248,rs61780894,rs12410166,rs1567402,rs68129642,rs74813748,rs72638577
CAPN8:rs68072917,rs4653877
CR1:rs55813295,rs55813295,rs55905875,rs55905875,rs55875400,rs55875400,rs56079743,rs55722040,rs56109143,rs56109143,rs56182593,rs56030162,rs56030162,rs56114117,rs56114117,rs56275712,rs55974817,rs55991702,rs55991702,rs55777427,rs55777427,rs56084806,rs56084806,rs55754610,rs56298042,rs56298042,rs56411060,rs55785804,rs55785804,rs55779204,rs55779204,rs56302981,rs56302981,rs55828967,rs55828967,rs56193300,rs56193300,rs56204135,rs56204135,rs56276874,rs56276874,rs55811516,rs56093932,rs56093932,rs56168197,rs56168197,rs56002956,rs56002956,rs55865820,rs55703877,rs55703877,rs55983342,rs55983342,rs55771013,rs55641872,rs55767627,rs55767627,rs56047029,rs56047029,rs56102197,rs56391032,rs56391032,rs55915510,rs55666812,rs55666812,rs55885741,rs55718069,rs55718069,rs55729318,rs55729318,rs56030390,rs56030390,rs55726190,rs55726190,rs55801279,rs55801279,rs56092428,rs56092428,rs55929821,rs56015412,rs56015412,rs1967828,rs56359850,rs56359850,rs55669141,rs55669141,rs55761008,rs55761008,rs56118781,rs56118781,rs55670331,rs55670331,rs56361204,rs56267956,rs55991765,rs55786257,rs55786257,rs55639135,rs55639135,rs55883797,rs56373231,rs56398864,rs56398864,rs55704769,rs56353950,rs56043167,rs56261321,rs56261321,rs55979404,rs56314797,rs56314797,rs55653509,rs56052213,rs56313533,rs56009212,rs56009212,rs55848422,rs56310405,rs56352571,rs56352571,rs56015758,rs56015758,rs56072537,rs56072537,rs56234734,rs55878655,rs55878655,rs56326394,rs56326394,rs55791318,rs55791318,rs55724640,rs55724640,rs55997834,rs55997834,rs56378827,rs56378827,rs55938003,rs55959625,rs56307287,rs56307287,rs55830750,rs55830750,rs55846619,rs55846619,rs55912924,rs55912924,rs55846728,rs56171829,rs56353976,rs56353976,rs55918401,rs55918401,rs55815626,rs55815626,rs56043430,rs56043430,rs56129138,rs56129138,rs55836426,rs55749808,rs55749808,rs55811653,rs55811653,rs56345001,rs55970375,rs55970375,rs55979222,rs55979222,rs55900657,rs55900657,rs55753551,rs55753551,rs56177984,rs56392313,rs56392313,rs55952684,rs56205102,rs55720721,rs55720721,rs55846136,rs55846136,rs56409218,rs56409218,rs55767030,rs55658501,rs56195127,rs56265707,rs56265707,rs55884405,rs55884405,rs55735142,rs56037822,rs56037822,rs56019948,rs56053139,rs55956260,rs55956260,rs55902061,rs55902061,rs55643045,rs55643045,rs56311501,rs56300774,rs56156766,rs55978552,rs55978552,rs55864105,rs55864105,rs55745723,rs55745723,rs55803443,rs55803443,rs55924525,rs56139167,rs56139167,rs55717512,rs55717512,rs56052895,rs56020776,rs56020776,rs55933005,rs55878543,rs55975235,rs55974321,rs55846628,rs55846628,rs56299420,rs56346880,rs56346880,rs55978988,rs55978988,rs56203358,rs56203358,rs56013449,rs56013449,rs55896092,rs56005541,rs56005541,rs55677167,rs55677167,rs56343686,rs56343686,rs55767641,rs55767641,rs55937128,rs55937128,rs55762770,rs55762770,rs56129265,rs56129265,rs55642106,rs55642106,rs55641633,rs56077723,rs56077723,rs56187018,rs56187018,rs56135198,rs56135198,rs56275361,rs56275361,rs56079149,rs56079149,rs56283013,rs56116155,rs56293185,rs56293185,rs56344090,rs56344090,rs56190053,rs56190053,rs56254726,rs56254726,rs55660220,rs55660220,rs56292493,rs56292493,rs55920254,rs55920254,rs56137601,rs56137601,rs55878564,rs55878564,rs55958312,rs56351037,rs56351037,rs56355024,rs56355024,rs55693072,rs56018236,rs55749945,rs56246301,rs56246301,rs55906506,rs55906506,rs55722809,rs55722809,rs55639201,rs55736256,rs56399315,rs56399315,rs55788979,rs55788979,rs55795011,rs56246645,rs56246645,rs55992341,rs55645336,rs55653580,rs56326826,rs55823564,rs55823564,rs55812785,rs55812785,rs56002218,rs55960704,rs55960704,rs56067147,rs56067147,rs56352598,rs56352598,rs55675707,rs55836902,rs55945035,rs55945035,rs55964020,rs56056140,rs55869469,rs55869469,rs56244656,rs56244656,rs55801565,rs56187514,rs55976152,rs55976152,rs56013080,rs56231283,rs56018255,rs55789578,rs55789578,rs55671111,rs55671111
CSRP1:rs34038448,rs80336819,rs12070807,rs515384
GCLM:rs2273406,rs2273407,rs34743713
HIVEP3:rs12135701,rs655198,rs78804462
NOTCH2NL:rs61815730,rs453773,rs2725962,rs2596007,rs384086,rs1690058,rs447289
UBE2T:rs34701328,rs55668607,rs14451,rs56283140,rs79811259
**Chromosome 2**

HK2:rs28362965
LONRF2:rs79560895,rs7575849,rs17023907,rs77359732,rs11902303
LYPD6B:rs79776678,rs7605478,rs6709479
PKP4:rs1110506
SCTR:rs6542532
TP53I3:rs36207095,rs36207096,rs72049316,rs35924313,rs36207097,rs3030953,rs34502732,rs34704159,rs72078174,rs72135009,rs34362184,rs5829915
TTC7A:rs79353883,rs12105384
WDR35:rs75004252,rs36061489
**Chromosome 3**
ADAMTS9:rs73832347,rs35802352,rs77195672
ADCY5:rs79462429
CACNA1D:rs79016258,rs78461250,rs75140300,rs77921094,rs17053128,rs76823338,rs75094150,rs72967859,rs78207451,rs11336778
FLNB:rs34911064,rs4453809,rs1718481,rs3772997,rs13067161
LPP:rs35902973,rs75330435,rs7653763,rs80236973,rs77602825,rs7646788
MAGI1:rs76005698
MYLK:rs7653257,rs7641367,rs820455
OSBPL10:rs35962535,rs79883182
PDCD6IP:rs75015608,rs71875147,rs28381975
PTPRG:rs28403395,rs588087,rs75480068,rs35061772,rs58646519,rs76704401,rs78836651
PXK:rs6775710,rs73835192
SEMA3B:rs12492880
SFMBT1:rs78698666
SGEF:rs74956401
SLC6A6:rs78003737
SUCLG2:rs74816918,rs75756678,rs79702177
**Chromosome 4**
ARHGAP10:rs11724772,rs7698520
HTT:rs71597206,rs16843836
LETM1:rs73202820
PALLD:rs76731571,rs2712149,rs2712148,rs2712147
SHROOM3:rs77486910,rs73828194
TBC1D9:rs57949768,rs74858974,rs71606900,rs7686941
TIFA:rs78320467,rs12510546
TSPAN5:rs75532697,rs62323584,rs7665200
**Chromosome 5**
ABLIM3:rs74471144
FBXO38:rs76818493
ISOC1:rs17163708
N4BP3:rs34062956,rs6601214,rs34226224,rs66991293,rs10635660
PDE4D:rs72530555,rs6450518,rs2702365,rs10616440
RGNEF:rs6881956,rs56692172,rs75553400,rs76642132,rs78248942
SLC22A5:rs274567,rs77123396

WWC1:rs76463523,rs75849226
ZNF346:rs2456181
**Chromosome 6**
CNKSR3:rs4870286,rs4870285,rs9478547,rs56359828,rs74679650
DOPEY1:rs74920300,rs78582064
MYB:rs17064301,rs11321182
RREB1:rs9392864,rs35178637
SYNJ2:rs78693252
TAF8:rs2493838,rs9394860,rs76562503,rs35711896
TPD52L1:rs17052846,rs55736347,rs76997203,rs3799757
**Chromosome 7**
ARPC1B:rs59680363
C7orf41:rs28701848,rs28364759,rs17547923,rs3750090
GCK:rs2908296,rs2908297
HIPK2:rs58760678,rs940543,rs77111783
RALA:rs35116603,rs71700028,rs74655958,rs72460287,rs70996821
SEMA3C:rs767733,rs35240493,rs58168478
**Chromosome 8**
ASAP1:rs17287702,rs73425525,rs77467207
DLGAP2:rs4875876,rs34951699,rs4875877,rs4875878
GATA4:rs10096189,rs17153694,rs10109678,rs78693845
NBN:rs13312849,rs1805844,rs72085652,rs76432414,rs1805799,rs71862785
NDRG1:rs71526285,rs61543350,rs73708719,rs75523276,rs13263811
PABPC1:rs73700221,rs56400641
**Chromosome 9**
RASEF:rs77598922,rs76617114
RXRA:rs62576308,rs12006409,rs79703336
SH3GLB2:rs5900824
UGCG:rs78807957
**Chromosome 10**
BAG3:rs12358294,rs196317,rs7894233,rs7909225,rs196316
CAMK1D:rs2768444,rs2768443
CTBP2:rs35602222,rs10510141,rs7080602
H2AFY2:rs56317707,rs35898392
PFKFB3:rs10905916,rs60080851,rs76884437,rs10795848
SEC61A2:rs9329337
**Chromosome 11**
ANO1:rs61885477
ARRB1:rs2507924,rs34867163
DHCR7:rs77926919,rs11233713
MARK2:rs76906610,rs928948,rs75857867
MPPED2:rs1222322,rs75642617,rs4922836
PHLDB1:rs75727057,rs5795129,rs67407552,rs35307519,rs33922258

PLEKHA7:rs4141194,rs76831006
RAB30:rs76931067,rs17144486,rs4944422
SPON1:rs1528668,rs1528669,rs73420271
STARD10:rs79843144
TCIRG1:rs4147783
**Chromosome 12**
CD9:rs17850834,rs11568275,rs11568274
CHPT1:rs59147798,rs74692062
DENND5B:rs34417003,rs62663961
ETV6:rs16907207,rs77355907,rs2710267,rs12816113
GAPDH:rs36015908,rs45612631,rs45459592,rs45564644
HSPB8:rs74648395
PITPNM2:rs71456785,rs61955220
SLC38A1:rs74831463,rs10880935,rs77006492,rs11183394
TPCN1:rs74907360
TUBA1A:rs58704368
VWF:rs78016141,rs2283332,rs1800375,rs1800376
**Chromosome 13**
FLT1:rs79452797,rs78305106,rs75363896
MCF2L:rs60864968,rs9549623,rs9549622
**Chromosome 14**
ACTN1:rs28657981,rs11626371,rs28514277,rs79000248,rs75442387,rs2268982
BDKRB2:rs56274409,rs61193624
C14orf43:rs79553653,rs76405516,rs28605040
CCDC88C:rs60137749,rs61988430
FBXO34:rs943591,rs943590,rs943592
PACS2:rs78224797,rs79894079
RAD51L1:rs7160794,rs76498298,rs79877406,rs73288074,rs76764799,rs35055159,rs1958112
SERPINA6:rs1998057,rs2281518,rs73346825
**Chromosome 15**
ABHD2:rs75057621,rs75080156,rs4566139,rs35226145
**Chromosome 16**
CBFA2T3:rs75269947,rs16965183,rs72140231,rs35191022,rs76188937,rs62045796,rs72815515,rs12445380
CHD9:rs35319530
FAM100A:rs1982784,rs76081350,rs78906334
KIAA0513:rs35349567,rs4991777,rs870149,rs870148,rs35649977
LCMT1:rs34551656,rs71385512
RAB11FIP3:rs74724425
RAB40C:rs62030903,rs62030902
ZFHX3:rs59660358,rs721823,rs74028152,rs35577902
**Chromosome 17**
ACE:rs13306085,rs4308,rs4309,rs72845022,rs59148455
AKAP1:rs74776597

IGFBP4:rs10305291
MAP3K14:rs77617592
MSI2:rs7214435,rs71365819,rs57563427,rs35327867,rs68081130,rs35611813,rs76341695,rs71387499,rs76089718
PITPNC1:rs8068510,rs1808693,rs7207677,rs79516805
PLCD3:rs10468559,rs7214439,rs7224944,rs73316962,rs77506254
SLC25A19:rs80337330,rs56090937
TBC1D16:rs67455669,rs59043351
**Chromosome 18**
ANKRD29:rs76162671,rs34580471,rs35841435,rs76695608
BCL2:rs3744949
C18orf1:rs74482571,rs73421368
EMILIN2:rs11660827,rs72870058
IMPA2:rs75061481,rs76907436
KLHL14:rs61737324,rs61737323
RAB31:rs8091795,rs78258961
SETBP1:rs79136715,rs16978187,rs9954058
**Chromosome 19**
ACTN4:rs973009,rs973010
CHST8:rs284327,rs1400615
DMPK:rs35500073,rs75890421
PTPRH:rs78703538,rs76990668
RGL3:rs34081
UPF1:rs9917075,rs34322276,rs73923167,rs80073951
ZC3H4:rs75022794,rs3745619,rs370649,rs442311,rs402833
**Chromosome 20**
PREX1:rs73326946,rs76652968,rs3746818,rs6095263,rs6063325,rs6019348,rs77571056,rs73911613,rs58812371
RPRD1B:rs79813465,rs6013782
SLC24A3:rs61185867,rs6106055,rs16980301,rs72073549,rs73282685
**Chromosome 21**
ICOSLG:rs35622408,rs75939338,rs2847224
NRIP1:rs72547880,rs7280064,rs11434871,rs66500136,rs78658187,rs35728511,rs34828787
PCBP3:rs2150454,rs55929196,rs78386080,rs75450717,rs63013260,rs7276623,rs76636709,rs2839025,rs34579989,rs72374338
**Chromosome 22**
CDC42EP1:rs11912966
CELSR1:rs75807774,rs74324720,rs9615971,rs79272284,rs5768827,rs9615969,rs76623479,rs5768828,rs71313051,rs5768750,rs9615970
MGAT3:rs75998268,rs78777985,rs34943118,rs9619814,rs9619815
MN1:rs75880328,rs74362945
MPST:rs5756489
SNAP29:rs1802503,rs5844445,rs165877,rs72465347,rs3202072,rs73879429,rs35888298,rs3041344,rs67020728
**Chromosome X**
SH3KBP1:rs35270010
